# Factors Related to Psychosocial Quality of Life for Children with Cerebral Palsy

**DOI:** 10.1155/2014/204386

**Published:** 2014-01-23

**Authors:** D. W. Tessier, J. L. Hefner, A. Newmeyer

**Affiliations:** ^1^Department of Family Medicine, College of Medicine, Ohio State University, 273 Northwood and High Building, 2231 North High Street, Columbus, OH 43201, USA; ^2^Developmental Pediatrics, Children's Hospital of the King's Daughters, Norfolk, VA, USA

## Abstract

*Background.* Current health services interventions focus on the treatment of the musculoskeletal impairments of cerebral palsy (CP). The goal of this study was to explore whether the severity of physical symptoms correlates with psychosocial quality of life (QOL) among pediatric patients with CP. *Methods.* A sample of 53 caregivers of children with CP was surveyed and health status information was extracted from patient medical records. Descriptive analysis explored the association between the main outcome variable, psychosocial QOL (CP QOL-child), and patient demographics, comorbidity (e.g., visual, hearing and feeding impairments, language delays, and epilepsy), CP severity (GMFCS), and the receipt of family centered care (MPOC-20). *Results.* Child psychosocial QOL decreased with increasing comorbidity but was not associated with CP symptom severity or any measured demographic factors. Reporting high levels of family centered care (FCC) was associated with higher psychosocial QOL in univariate analysis but was not significant when controlling for comorbidities. *Conclusion.* There is no clear connection between symptom severity and psychosocial QOL in children with CP. Comorbidity however is strongly associated with psychosocial QOL. Focusing on reducing CP comorbidities could have a positive impact on psychosocial QOL.

## 1. Introduction

Cerebral palsy (CP), with a prevalence of 1 in 278 children, is the most common childhood motor impairment [1]. The term “cerebral palsy” describes a group of permanent disorders of the development of movement and posture attributed to nonprogressive disturbances that occurred in the developing fetal or infant brain [2]. There are no specific treatments for the brain insults that lead to the motor dysfunction of CP. Instead, treatment is focused on a range of therapies (e.g., physiotherapy, surgery, medication) with the aim of enhancing a patient's overall quality of life by mitigating musculoskeletal impairments and pain [3, 4]. Health-related quality of life (QOL) is a subdomain of QOL that encompasses physical, mental, and social well-being [4–6]. For children with CP, health-related QOL has been increasingly positioned as an important outcome indictor [6, 7].

Research indicates that pediatric and adolescent patients with CP have impaired functional and psychosocial QOL when compared with their normative peers [8–10]. While traditional CP treatments have been associated with improvements to patients' functional quality of life, these treatments have had less impact on psychosocial QOL [3, 11, 12]. Specifically, previous studies have found a weak relationship between measures of CP severity and psychosocial QOL [7, 13–15]. This has led to a call for further examination of other patient level variables that could influence psychosocial QOL [15]. In the pediatric CP literature, highly prevalent dysfunctions accompanying the primary diagnosis of musculoskeletal impairment include visual, hearing and feeding impairments, language delays, and epilepsy [16, 17]. The cooccurrence of many of these impairments, that is, comorbidities, may be associated with decreased QOL [16]. If this is indeed the case, clinical interventions which focused on reducing CP comorbidities could have a positive impact on patient QOL.

Additionally, there is a need to explore mechanisms beyond clinical interventions to improve psychosocial QOL for patients with CP. Children with CP have complex healthcare needs and rely heavily on the healthcare system, spending 2.2 times the amount of time in outpatient care settings when compared with their healthy peers [18], and often interacting with multiple care providers [19]. This high utilization of the health and related services positions the health delivery system as a potential focus of psychosocial QOL interventions. One such intervention is family centered care, considered the best approach to service delivery in pediatric rehabilitation [20–22]. Family centered care (FCC) is a set of principles underlying care delivery that recognizes the role of the family in the child's care, focusing on shared decision making, information sharing, empowerment, and care coordination to enhance the quality of life for all family members [20].

We provide a preliminary exploration of the role of comorbidities and family centered care on QOL in children with CP by assessing two specific aims: (1) to assess if the perceived psychosocial QOL of a child with CP is associated with patient demographics and health status, including CP severity or comorbidity. Prior research has found a strong correlation with physical QOL but the association with psychosocial QOL is less clear [7, 13–15]. Given this, we hypothesize that patient psychosocial QOL will not be associated with severity and comorbidity; (2) to determine if there is an association between the receipt of family centered care and psychosocial QOL for these children. Based on the few studies that have found a positive association in pediatric populations of children with disabilities [20, 22–25], we hypothesize that among a sample of pediatric patients with CP reporting high levels of family centered care will be associated with increasing psychosocial QOL.

## 2. Methods

### 2.1. Study Population and Survey Protocol

To achieve these aims, a sample of caregivers of children with CP who attended outpatient CP and therapy clinics at a large tertiary care University Medical Center was surveyed regarding their experiences with the health care system. Demographic and health status information was extracted from the medical records of the respondents' children. Specifically, subjects with CP were identified through a review of the electronic medical records of the Developmental Disabilities Clinic, the Cerebral Palsy Clinic, and the Physical Therapy/Occupational Therapy clinics at the Children's Hospital. Caregivers of patients identified as having cerebral palsy in the electronic medical record were recruited for participation by a member of the research team during their clinic or therapy appointments, prior to clinical evaluation. Caregivers who agreed to participate completed a consent form, and verbal assent was obtained from pediatric patients when appropriate. Caregivers were then provided a paper and pencil survey. There were no specific clinical exclusion criteria. This study protocol was approved by the Nationwide Children's Hospital IRB.

### 2.2. Variables

The caregiver survey included a Measure of Processes of Care-20 (MPOC-20) and the parent-proxy Cerebral Palsy Quality of Life Questionnaire for Children (CP QOL-Child). The MPOC-20 is a self-report measure of parents' perceptions of the extent to which the health services the family receives are family centered, measured on a seven point scale across five domains with a higher score indicating a higher level of family centered care [25]. In large validation studies of caregivers of children with CP this measure has been demonstrated to be valid and reliable with a positive correlation with standardized measures of satisfaction [25–27].

The CP QOL-Child is a condition specific measure of QOL for children with CP. The parent-proxy version covers two psychosocial domains: “social well-being and acceptance” and “emotional well-being and self-esteem” [28]. To score the CP QOL-Child, caregiver's responses are aggregated across items and an average is calculated across the sample and then converted to a 0 to 100 scale based on the coding algorithm provided by Waters et al. [29]; a higher average score equals higher QOL. In multiple validation studies the CP QOL-Child had good test-retest reliability, construct validity and internal consistency, and Cronbach's alphas of 0.74 to 0.91 [5, 28, 30, 31]. Additionally, parental perceptions provided in the CP QOL-Child correlate with medical professional ratings of patient QOL for children with CP [32]. There is a version of the CP QOL-child that is self-report for children 9 to 12 years old. However, only the parent-report version was used in this study because the sample is between 2 and 12 years and not all children in the 9–12 age group would have been able to complete the CP-QOL independently due to cognitive and communication limitations.

A retrospective chart review of all participating patients was conducted to extract patient age, race, sex, severity of illness, and level of comorbidity. For the purposes of analysis age was coded as a continuous variable, race was categorical “white” and “nonwhite” (a necessity given the sample size), and sex was binary male or female. Severity of illness was operationalized by the Gross Motor Function Classification System (GMFCS) [33]. A patient's GMFCS is assigned by the physician as I through V, with V as the most severe impairment in gross motor function during the conduct of daily activities. A comorbidity is recorded in the patient's chart for each impaired organ system and physical or mental disability (ENT, skin, eye/head, cardiology, etc., and also documented language disorder, developmental delay). This categorization scheme for comorbidities differs from the traditional count of medical diagnoses but is consistent with the pediatric cerebral palsy literature in which highly prevalent dysfunctions accompanying the primary diagnosis of musculoskeletal impairment include visual, hearing and feeding impairments, language delays, and epilepsy [16, 17]. For the purposes of analysis comorbidity is coded as “none,” “one”, or “two or more.”

### 2.3. Analysis

First, survey measures were scored and results entered into a password-protected MS Excel research database. Next, the patient health and sociodemographic variables extracted from health records were appended to the survey data using unique patient identifiers. Means were then calculated for all variables and a series of univariate linear regressions of the demographic, severity, and family centered care (MPOC-20) variables on the two psychosocial QOL domains was conducted. When more than one variable was significantly associated with a QOL domain these variables were put into a multivariate linear regression to assess independent associations. Regression diagnostics were assessed to determine if the residuals were normally distributed. Data analysis was completed using STATA 12.0 [34].

## 3. Results and Discussion

### 3.1. Results

The survey and data collection from patient medical records was completed for 53 children with a diagnosis of CP. The sample's demographic information is presented in Table 1. Children were majority white, between the ages of 2 and 12 and functioning across all GMFCS levels, with 80% having one or more comorbidities.

Table 2 presents the mean scores for the MPOC-20 and QOL domains. Mean scores on the five MPOC domains ranged from 4.4, “providing general information” to 5.9, “respectful and supportive care” (on a scale of 1 to 7 with a higher score denoting a high level of family centered care). The mean QOL score for the social well-being and acceptance scale was 78.7 with a standard deviation of 13.2 (with 100 being the highest possible score). The mean score for emotional QOL was 82.5 with a standard deviation of 13.5.

Table 3 presents a series of univariate linear regressions of the sociodemographic variables, severity variables, and level of family centered care on both psychosocial QOL domains. No significant association was found between age, sex, or race and either psychosocial QOL domain. GMFCS level was also not significantly associated with psychosocial QOL. Comorbidity was strongly correlated with both social well-being and emotional well-being. Specifically, an additional comorbidity is associated with a 6-point decrease on the social well-being scale (*P* = 0.016) and a 9-point decrease on the emotional well-being scale (*P* = 0.000). The only MPOC domain associated with either psychosocial QOL domain was care coordination, a step up the seven-point scale toward higher family centered care was associated with a 4.3-point increase in emotional well-being and self-esteem (*P* = 0.025).

In a multivariate linear regression model an additional comorbidity was independently associated with a 9-point decrease in this QOL score (*P* = 0.001). Controlling for level of comorbidity, the MPOC measure of care coordination was no longer significantly associated with psychosocial QOL (*P* = 0.107). The adjusted R-squared of this model is 0.26 meaning that 26% of the variation in emotional well-being and self-esteem is explained by the two variables included in this model. Regression diagnostics show that the residuals are normally distributed, based on visual analysis of a stem and leaf plot of the studentized residuals and a k-density plot. The other patient and MPOC variables were not included in this model because none were significantly associated with either QOL domain in the univariate regressions. No multivariate model was conducted for the QOL domain social well-being because the only measured variable associated with this domain in univariate analyses was level of comorbidity.

### 3.2. Discussion

Among a sample of pediatric patients with CP, child psychosocial QOL decreased with increasing comorbidity but was not associated with CP symptom severity. To our knowledge this is the first study to explore the relationship between comorbidity and QOL in the pediatric CP population. While it has been shown that surgery and pain management, the focus of traditional CP interventions, can improve a child's functional quality of life, rarely does their psychosocial QOL improve as well [3, 11, 12, 14, 35]. Arnaud et al. [14] propose that perhaps social interactions, school environment, and other social factors play a larger role in determining a child's psychosocial quality of life. Based on the descriptive results of the present study future research should evaluate if comorbidity plays a critical role in shaping these social interactions. Perhaps children with multiple comorbidities and thus high care complexities are either not able to engage in age appropriate social interactions due to the complexity of their cases or are not afforded the opportunity to do so, driving the strong association between increasing comorbidity and decreasing psychosocial QOL.

The second aim of this research was to explore the potential impact of receipt of family centered care on the QOL of the child with CP, with a specific focus on psychosocial QOL. We did not find support for the a priori hypothesis that reporting high levels of family centered care is associated with increasing psychosocial QOL. While one measure of family centered care, providing care coordination assistance, was significantly associated with both psychosocial QOL domains, in a multivariate model controlling for level of comorbidity this measure of family centered care was no longer significant. This finding is counter to a previous study which found an association between family centered care and family quality of life for families of young children with disabilities [24]. Additionally, in a national sample of caregivers of children with CP, the perception of receiving a high level of FCC was associated with having community, financial, and social support needs met [23].

The discrepancy between the present results and previous research may be due to a combination of two factors: (1) the prior studies lack multivariate modeling that controls for comorbidity and (2) the present study focused on child quality of life and previous studies focused on adequate support for the family. Theoretically, these family variables may be a pathway through which FCC positively affects child psychosocial QOL, but the relationship was not strong enough to detect in our study given the limited sample size. Future research should compare the psychosocial QOL of children in different care settings to further investigate family centered care and the potential role of family support as a mediating variable.

### 3.3. Limitations

An important limitation of this study is the descriptive study design and the small, convenience sample. However, this study is an important step in the assessment of potential health services interventions that can improve psychosocial QOL. This is also the first study, to our knowledge, to consider health status variables beyond GMFCS levels. The finding that comorbidity is significantly associated with psychosocial QOL highlights an important area of future study. This descriptive study should lead to future research in large patient populations across care settings.

Another important limitation is the use of the parent-proxy CP-QOL to assess child QOL. It has been demonstrated that children with CP generally report higher QOL scores than their parents [36]. If the bias is uniform across parental respondents the relationship between variables would not be affected. However, there could be results affected if the magnitude of this discrepancy varies by patient or family characteristics. For example, a previous study found associations between parental stress level and proxy-reported QOL. Future larger scale studies could explore these potential confounders and control for parental stress level when proxy-report is necessary due to the age of the study population.

## 4. Conclusions

For children with CP, QOL has been increasingly positioned as an important outcome indictor [6, 7]. Given this, the identification of associated variables amenable to intervention has important practice implications. Specifically, the finding of a strong association between comorbidity and psychosocial QOL positions comorbidity as an area of intervention that could have a positive impact on patient QOL. In the present study comorbidity was defined as a physical or mental impairment beyond musculoskeletal dysfunction (e.g., including impairments in hearing, sight, epilepsy, and language or developmental delay). Interventions to reduce comorbidity may not eliminate intractable diagnoses but could focus on reducing the impact of a patient's impairment and potentially reduce the number of disabilities that accompany the musculoskeletal challenges of CP. The findings of this study show that interventions which focused on this goal may lead to improvements in pediatric CP patients' psychosocial QOL.

## Figures and Tables

**Table 1:** Demographic characteristics and health status of a sample of children with cerebral palsy.

Variable	*N*	%
Total sample	53	100
Age		
2–5	27	50.9
6–12	26	49.1
Sex		
Male	27	50.9
Female	26	49.1
Race		
White	45	84.9
Minority	8	15.1
CP symptom severity (GMFCS)		
I	15	28.3
II	5	9.4
III	3	5.7
IV	8	15.1
V	5	9.4
Missing data^a^	17	32.1
Comorbidities		
0	11	20.8
1-2	26	49.1
>2	16	30.1

^a^GMFCS was pulled from the notes field of each patient chart and could not be ascertained for 17 participants.

**Table 2:** Mean scores for MPOC-20 and QOL domains.

Variable	Mean	STDEV
MPOC: enabling and partnership	5.7	1.1
MPOC: providing general information	4.4	1.8
MPOC: providing specific information about the child	5.5	1.2
MPOC: coordinated and comprehensive care	5.7	1.0
MPOC: respectful and supportive care	5.9	0.9
QOL: Social well-being and acceptance	78.7	13.2
QOL: emotional well-being and self-esteem	82.5	13.5

**Table 3:** Univariate linear regressions of each individual patient characteristic and MPOC score on each of the two psychosocial QOL variables.

Independent variables	Dependent variables			
	QOL: social well-being and acceptance		QOL emotional well-being and self-esteem	
	Coef.	*P* value	Coef. (*P* value)	*P* value
Comorbidity	−**5.92**	0.016	−**8.89**	0.000
Severity	−0.72	0.631	−2.28	0.131
Age	0.205	0.740	0.142	0.828
Sex	3.56	0.318	−0.28	0.941
Race	−2.63	0.600	−7.24	0.167
MPOC: enabling and partnership	1.71	0.291	1.73	0.320
MPOC: providing general information	0.75	0.473	1.61	0.147
MPOC: providing specific information	1.53	0.261	2.28	0.119
MPOC: coordinated care	2.40	0.187	**4.30**	0.025
MPOC: respectful and supportive	1.74	0.381	3.77	0.075

Note: bold denotes a significant association at *P* < 0.05.
